# Dialectical Behavior Therapy for Patients With Bipolar Disorder: A Systematic Review and Meta-Analysis

**DOI:** 10.1192/j.eurpsy.2025.644

**Published:** 2025-08-26

**Authors:** A. L. L. Larcipretti, J. R. de Souza, B. A. D. A. Rocha, J. B. da Silva Neto, F. C. Gomes, M. Y. Ferreira, G. C. Carpi, L. Baldaçara, A. G. da Silva

**Affiliations:** 1 School of Medicine, Federal University of Ouro Preto, Ouro Preto; 2 School of Medicine, Universidade Federal do Rio de Janeiro, Rio de Janeiro; 3 Laboratory of Digital Psychiatry, Universidade Federal do Paraná, Paraná; 4 Faculty of Medicine, Federal University of Minas Gerais, Belo Horizonte, Brazil; 5 Department of Neurosurgery, Lenox Hill Hospital, New York, United States; 6 Pediatrics, Hospital de Clinicas de Porto Alegre, Porto Alegre; 7 School of Medicine, Universidade Federal do Tocantins, Tocantins; 8 psychiatry, Associação Brasileira de Psiquiatria, Rio de janeiro, Brazil; 9 Faculty of Medicine, Universidade do Porto, Porto, Portugal

## Abstract

**Introduction:**

Dialectical Behavior Therapy (DBT) is a comprehensive evidence-based psychotherapy that focuses on teaching skills regarding the acceptance of circumstances and coping with emotional responses.

**Objectives:**

The objective of this study is to perform a systematic review and meta-analysis to investigate the efficacy of DBT in patients diagnosed with Bipolar Disorder (BD) in order to ascertain whether it improves the recurrence of manic and depressive symptoms.

**Methods:**

A systematic search of the PubMed (MEDLINE), Embase, Web of Science, and Cochrane Library databases was conducted to identify studies that had applied DBT to patients with a diagnosis of BD.

**Results:**

A total of 343 patients were included in the study, comprising participants from eight randomized and non-randomized trials. Of whom, 196 patients (57.1%) underwent DBT and pharmacological treatment, while 147 patients (42.9%) were treated with alternative interventions. A total of 12 to 36 sessions of DBT were conducted across all trials, with a follow-up period ranging from three to 15 months. The age range of the participants was 15.8 to 49.3 years. All studies included patients diagnosed with BD type I (n=175), five articles included patients with BD type II (n=100), and two included patients with BD-NOS (Not Otherwise Specified) (n=68). The primary endpoint analyzed was the mean change in the Beck Depression Inventory-II (BDI-II), as reported by three of the included studies. The meta-analysis yielded no statistically significant results, with a mean difference of -4.49 (95% CI: -11.75, 2.76; I² = 6%; p = 0.22) (Figure 2). The analysis of the Young Mania Rating Scale (YMRS) revealed a mean of 5.96 in a total of 72 patients (95% CI: 0.29-11.64; I² = 97.39%; p < 0.001) (Figure 3).

**Image 1:**

**Figure:**
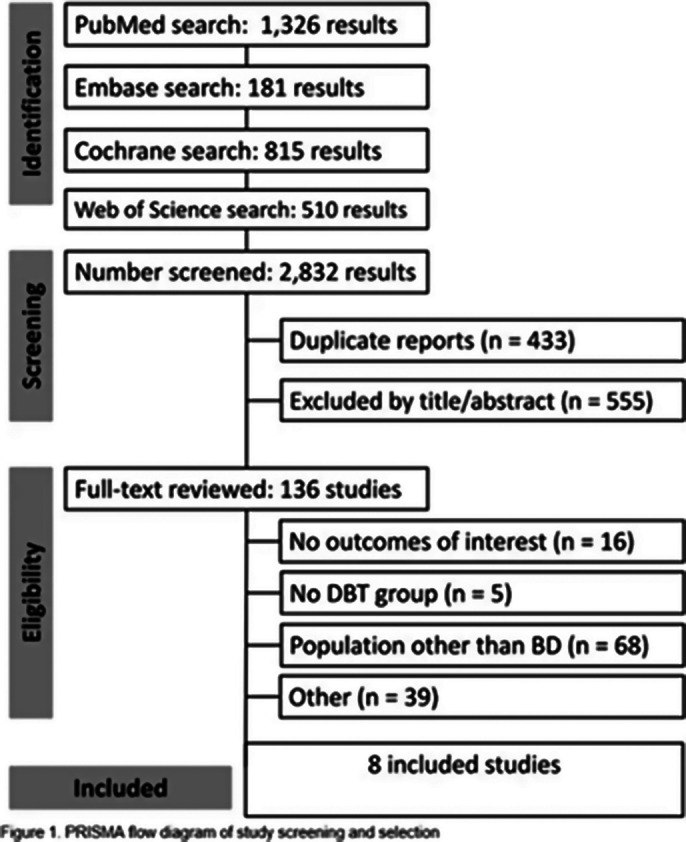

**Image 2:**

**Figure:**
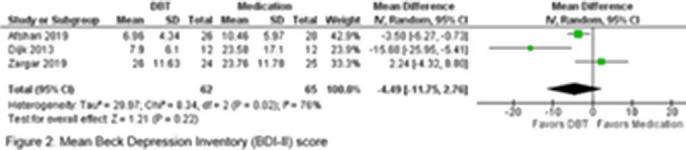

**Image 3:**

**Figure:**
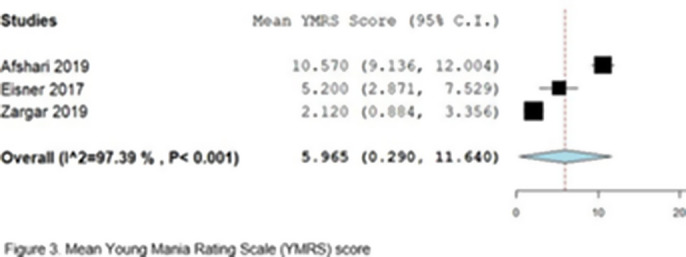

**Conclusions:**

The DBT was observed to have a beneficial impact on mood episodes and symptomatic manifestations among adolescents and adults diagnosed with BD. Therefore, it may be postulated that the DBT could be employed in conjunction with pharmacological agents to mitigate the severity of symptoms and enhance the overall quality of life in patients with such a diagnosis.

**Disclosure of Interest:**

None Declared

